# The Effect of Varying the Amount of Short Hemp Fibers on Mechanical and Thermal Properties of Wood–Plastic Composites from Biobased Polyethylene Processed by Injection Molding

**DOI:** 10.3390/polym14010138

**Published:** 2021-12-30

**Authors:** Celia Dolçà, Eduardo Fages, Eloi Gonga, David Garcia-Sanoguera, Rafael Balart, Luis Quiles-Carrillo

**Affiliations:** 1 Textile Industry Research Association (AITEX), Plaza Emilio Sala, 1, 03801 Alcoy, Spain; cdolza@aitex.es (C.D.); efages@aitex.es (E.F.); egonga@aitex.es (E.G.); 2 Technological Institute of Materials (ITM), Universitat Politècnica de València (UPV), Plaza Ferrándiz y Carbonell, 1, 03801 Alcoy, Spain; dagarsa@dimm.upv.es (D.G.-S.); rbalart@mcm.upv.es (R.B.)

**Keywords:** BioHDPE, green composites, hemp short natural fibers, non-isothermal crystallization, mechanical properties

## Abstract

Biobased HDPE (bioHDPE) was melt-compounded with different percentages (2.5 to 40.0 wt.%) of short hemp fibers (HF) as a natural reinforcement to obtain environmentally friendly wood plastic composites (WPC). These WPC were melt-compounded using a twin-screw extrusion and shaped into standard samples by injection molding. To improve the poor compatibility between the high non-polar BioHDPE matrix and the highly hydrophilic lignocellulosic fibers, a malleated copolymer, namely, polyethylene-*graft*-maleic anhydride (PE-g-MA), was used. The addition of short hemp fibers provided a remarkable increase in the stiffness that, in combination with PE-g-MA, led to good mechanical performance. In particular, 40 wt.% HF drastically increased the Young’s modulus and impact strength of BioHDPE, reaching values of 5275 MPa and 3.6 kJ/m^2^, respectively, which are very interesting values compared to neat bioHDPE of 826 MPa and 2.0 kJ/m^2^. These results were corroborated by dynamic mechanical thermal analysis (DMTA) results, which revealed a clear increasing tendency on stiffness with increasing the fiber loading over the whole temperature range. The crystal structure was not altered by the introduction of the natural fibers as could be seen in the XRD patterns in which mainly the heights of the main peaks changed, and only small peaks associated with the presence of the fiber appeared. Analysis of the thermal properties of the composites showed that no differences in melting temperature occurred and the non-isothermal crystallization process was satisfactorily described from the combined Avrami and Ozawa model. As for the thermal degradation, the introduction of HF resulted in the polymer degradation taking place at a higher temperature. As for the change in color of the injected samples, it was observed that the increase in fiber generated a clear modification in the final shades of the pieces, reaching colors very similar to dark woods for percentages higher than 20% HF. Finally, the incorporation of an increasing percentage of fibers also increased water absorption due to its lignocellulosic nature in a linear way, which drastically improved the polarity of the composite.

## 1. Introduction

Nowadays, polymers constitute a basic aspect of our daily lives. Their use is widely extended in different essential sectors for society, such as packaging production for food and farming industries [1,2]. Their application is very extended due to their high versatility, durability and their low cost. As a result of their widespread use, there are great amounts of wastes that must be correctly managed by mechanical recycling processes to avoid that those wastes end up in the rubbish dump [3,4]. The United Nations has proposed several measures in their sustainable development 2030 agenda to improve sustainability and natural resources exploitation [5]. As an alternative to the high dependence on petroleum of most of the current polymers, the use of biobased polymers is proposed [6]. As a result, the production of biobased polymers is currently in a growing state. In 2018, 2.11 million tons were produced, and 2.62 million tons are expected to be produced in 2023 [7]. These biobased polymers can be obtained from different natural resources. Some of the most well-known polymers are usually obtained from lignocellulosic sources, such as thermoplastic starch (TPS); polylactic acid (PLA), which is produced from lactic acid coming from corn; or polyhydroxyalkanoates (PHA), which are obtained through fermentation of a carbon-rich source [8]. In addition to those polymers, which are highly biodegradable, there exist non-biodegradable alternatives, such as bio-PE produced from bioethanol obtained from sugar cane [9]. Bio-PE arises as an interesting alternative to environmental problems, which is related to the fact that it possesses the same properties as its petrochemical counterpart [10]. This gives bio-PE a great advantage against other biopolymers due its intrinsic properties, which include a good mechanical behavior, electrical resistance, thermal stability, permeability and chemical resistance. These properties have made bio-PE one of the most used in the industrial field [11,12,13]. An example the mechanical properties of BioHDPE can be seen in the work of García et al., who reported a tensile strength of 19.5 MPa and an elongation at break of 500% [14]. 

Together with the use of new polymers from renewable resources, the interest in natural fiber reinforced plastics (NFRP) [15,16,17] and wood plastic composites (WPC) [18,19,20] has remarkably increased. The traditional lack of environmental concern led to a still active linear economy system. This system requires the obtention of resources for the fabrication of products that will be discarded at the end of their useful life [21]. In this context, the revalorization of byproducts from industries or agroforestry is being applied to obtain WPC, aligning with the principles of the circular economy concept (CE). The main objective of circular economy is to establish a loop in which wastes can be reused to obtain new products. In this sense, the need for raw materials can be reduced, as well as the necessary energy for their obtention [22,23]. Nowadays, composites with 70 wt.% content in wood-based fillers and 30 wt.% of polymer content have been developed in order to maximize the environmentally friendly impact of reusing a waste. However, the introduction of fillers over 40 wt.% makes the fluidity of the materials to drastically decrease, which implies a difficulty to produce those materials by techniques such as injection molding. It is for this reason that filler contents in composites are considerably lower [24]. Apart from the amount of filler used, the type of filler and their shape and size also determine their final properties. In general terms, if the particles are thin and long, better mechanical properties are obtained [25,26]. Particularly, vegetal fibers can play an important role in the development of biodegradable composites with enhanced mechanical properties. Moreover, they can solve the current environmental problems of composites, especially the ones related to the recyclability of glass fiber reinforced plastics (GFRP).

This geometry of particle can be obtained from natural micronized fibers. It is for this reason that lots of works have been done where the resistance of different polymers has been increased through the incorporation of fibers that come from pine wastes of husk fibers, among others [27,28]. One of the most interesting fibers for the obtention of WPC is raw hemp (*Cannabis sativa* L.), which is a waste from the agricultural industry with great availability [29]. This great availability makes hemp fibers cheap [30], which make this waste interesting due to its great ability to reduce the cost of the polymer where it is introduced [31]. Furthermore, the addition of hemp can enhance the mechanical properties of the composite due to its morphology. Nonetheless, it is necessary to improve the affinity between hemp and the polymer through compatibilization [32]. Their intrinsic lack of compatibility is due to the hydrophilic behavior of the fibers, which is different from the hydrophobic nature of the polymer. To solve this problem, there exist several procedures that have successfully improved the interaction between both compounds. These processes include the treatment of the fibers, for example, with plasma, or the use of compatibilizers [33].

The use of compatibilizers is especially interesting, as it is not necessary to make complex treatments to the fillers. This strategy is based on the introduction of modified polymeric chains through a grafting process. By this methodology PE-g-MA can be obtained, which has been used to successfully obtain lignocellulosic-based composites. This is ascribed to the fact maleic anhydride (MA) groups in PE-g-MA can react with hydroxyl groups in lignocellulosic wastes, acting as chemical bonds with the polymer [11]. In this sense, Roumeli et al. compared the mechanical performance of hemp composites with their non-compatibilized counterparts. In general, the mechanical response considerably improved in the compatibilized materials, presenting an improvement of up to 70% in the resistance of the blends with the highest filler content [34]. As a result of the investigations made on WPC, it is possible to produce components with a wide range of applications such as indoor and outdoor furniture like benches or fences, fabrication of automotive parts, building structures or coatings, among others [35,36].

This work is centered in the obtention of highly efficient environmental materials through injection molding processes introducing different proportions of hemp fiber into a BioHDPE matrix. The main objective has been to produce WPC with a high proportion of short hemp fibers to make the blends more environmentally friendly. PE-g-MA has been used as a compatibilizer to improve the fiber–polymer interaction. These materials allow to provide the fibers with an added value, reducing the cost of the BioHDPE-based composites. Mechanical, morphological, thermal and thermomechanical properties, among others, have been evaluated to thoroughly analyze how the increase in hemp fiber content affects the final properties of the composites. The developed materials will be made of a biobased matrix and lignocellulosic fillers.

## 2. Materials and Methods

### 2.1. Materials

BioHDPE was provided as SHA7260 by FKuR Kunststoff GmbH (Willich, Germany) and manufactured by Braskem (São Paulo, Brazil). The manufacturer supplies this green polyethylene in pellets with a density of 0.955 g/cm^3^ and a melt flow index (MFI) of 20 g/10 min, measured with a load of 2.16 kg and a temperature of 190 °C.

The copolymer polyethylene-graft-maleic anhydride (PE-g-MA) with CAS Number 9006-26-2 and MFI values of 5 g/10 min (190 °C/2.16 kg), was obtained from Sigma-Aldrich S.A. (Madrid, Spain). This PE-based copolymer was selected due to its dual functionality (polar/non-polar). The proportion of maleic anhydride (MA) in the copolymer is 0.5 wt.% according to the supplier. In relation to the compatibilizer, in order to obtain a balanced proportion and to maintain what has been analyzed in previous studies, 10% PE-*g*-MA was added in relation to the amount of fiber.

**Figure 1:**
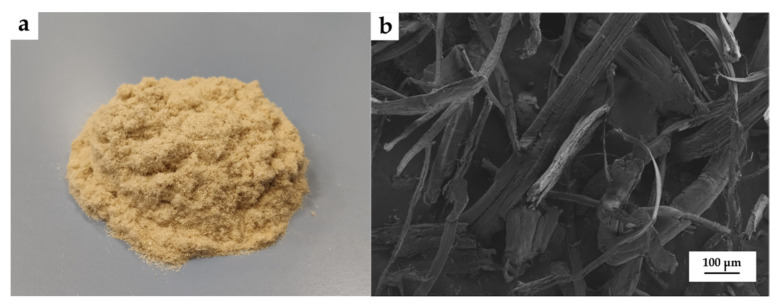
(**a**) Visual aspect and (**b**) field emission scanning electron microscopy (FESEM) images at 100x of Hemp fibers.

Hemp fiber was supplied by SCHWARZWÄLDER TEXTIL-WERKE (Schenkenzell, Germany). This fiber showed an irregular rough shape with an average coarseness of 15–50 μm, specific gravity 1.48–1.50 g/cm^3^ and elongation at break of 1.3%. Figure 1 shows the visual appearance of the hemp fiber. 

### 2.2. Sample Preparation

Fibers and BioHDPE were dried at 60 °C for 48 h in an MDEO dehumidifier dryer (Industrial Marsé, Barcelona, Spain) to remove any residual moisture prior to processing. Both were pre-mixed in a zip bag. The materials were then fed into the main hopper of a co-rotating twin-screw extruder from Construcciones Mecánicas Dupra, S.L. (Alicante, Spain). This extruder has a screw diameter of 25 mm with a length-to-diameter ratio (L/D) of 24. The extrusion process was carried out at 25 rpm, setting the temperature profile, from the hopper to the die, as follows: 140–145–150–155 °C. The different bioHDPE composites were extruded through a round die to produce strands and, subsequently, pelletized using an air-knife unit. In all cases, residence time was approximately 1 min. Table 1 summarizes the compositions and coding.

The compounded pellets were shaped into standard samples by injection molding in a Meteor 270/75 from Mateu & Solé (Barcelona, Spain). The temperature profile in the injection molding unit was 140 °C (hopper), 150 °C, 155 °C and 160 °C (injection nozzle). A clamping force of 75 tons was applied while the cavity filling and cooling times were set to 1 and 10 s, respectively. Standard samples for mechanical and thermal characterization with an average thickness of 4 mm were obtained.

### 2.3. Material Characterization

#### 2.3.1. Mechanical Tests 

Tensile tests were carried out in a universal testing machine ELIB 50 from S.A.E. Ibertest (Madrid, Spain) using injection-molded dog bone-shaped samples as indicated by ISO 527-1:2012. A 5 kN load cell was used and the cross-head speed was set to 5 mm/min. In order to improve the mechanical characterization, shore hardness was measured in a 676-D durometer from J. Bot Instruments (Barcelona, Spain), using the D-scale, on injection-molded samples with dimensions 80 × 10 × 4 mm^3^, according to ISO 868:2003. Toughness was also studied on injection-molded rectangular samples with dimensions of 80 × 10 × 4 mm^3^ by the Charpy impact test with a 1-J pendulum from Metrotec S.A. (San Sebastián, Spain) on notched samples (0.25 mm radius v-notch), following the specifications of ISO 179-1:2010. All tests were performed at room temperature, that is, 25 °C, and at least 5 samples of each material were tested, and their values averaged.

#### 2.3.2. XRD Properties

XRD patterns were registered at room temperature using KRISTALLOFLEX K 760-80F equipment, operating at voltage of 40 kV and −40 mA. The applied radiation from target Cu Kα was nickel filtered (λ = 0.154 nm). The range of scattering angles (2θ) was 5 º to 60 ° with a step size of 0.05 º and a speed of 1 º/min.

#### 2.3.3. Morphology

The morphology of the fracture surfaces of the BioHDPE-natural fiber composites, obtained from the impact tests, was observed by field emission scanning electron microscopy (FESEM) in a ZEISS ULTRA 55 microscope from Oxford Instruments (Abingdon, UK), working at an acceleration voltage of 2 kV. Before placing the samples in the vacuum chamber, they were sputtered with a gold-palladium alloy in an EMITECH sputter coating SC7620 model from Quorum Technologies, Ltd. (East Sussex, UK). 

#### 2.3.4. Thermal Analysis

In order to obtain the main thermal transitions of BioHDPE-hemp fiber composites were obtained by differential scanning calorimetry (DSC) in a Mettler-Toledo 821 calorimeter (Schwerzenbach, Switzerland). An average sample weight ranging from 5 to 7 mg was subjected to the following three-stage dynamic thermal cycle: first heating from 20 °C to 160 °C followed by cooling to 0 °C, and second heating to 250 °C. Heating and cooling rates were set to 10 °C/min. All tests were run in nitrogen atmosphere with a flow rate of 66 mL/min using standard sealed aluminum crucibles (40 μL). The degree of crystallinity ($\chi_{c}$) was determined following the Equation (1):

(1) $$\chi_{c}\left(\%\right)=\left[\frac{\Delta H_{m}}{\Delta H_{m}^{0}\cdot \left(1-w\right)}\right]\cdot 100$$

where $\Delta H_{m}$ (J/g) stands for the melting enthalpy of the sample; $\Delta H_{m}^{0}$ (J/g) represents the theoretical melting enthalpy of a fully crystalline BioHDPE, that is, 293.0 J/g [37]; and w corresponds to the weight fraction of different fibers in the formulation.

Thermogravimetric analysis (TGA) was performed in a LINSEIS TGA 1000 (Selb, Germany). Samples with an average weight between 15 and 25 mg were placed in standard alumina crucibles of 70 µL and subjected to a heating program from 30 °C to 700 °C at a heating rate of 10 °C/min in air atmosphere. The first derivative thermogravimetric curves (DTG) were also determined, expressing the weight loss rate as the function of time. All tests were carried out in triplicate.

The crystallization model used to describe the behavior of the BioHDPE/hemp composites is the one proposed by Liu et al., which combines with the model of Avrami and Ozawa in Equation (2) [38]:

(2) logϕ=logF(T)−blogt

where ϕ is indicative of the cooling speed used during the test and t is the necessary time to reach a certain degree of crystallinity. The obtention of the parameters of the model was carried out plotting logϕ vs. logt, taking time values for different relative crystallinity degrees (20%, 40%, 60% y 80%) and different cooling velocities (5 °C/min, 10 °C/min, 15 °C/min y 20 °C/min).

#### 2.3.5. Thermomechanical Characterization

A DMA1 dynamic analyzer from Mettler-Toledo (Schwerzenbach, Switzerland) was used for dynamic mechanical thermal analysis (DMTA). Injection-molded samples with dimensions of 20 × 6 × 2.7 mm^3^ were subjected to a dynamic temperature sweep from −150 °C to 130 °C at a constant heating rate of 2 °C/min, a frequency of 1 Hz and a maximum cantilever deflection of 10 µm. All tests were carried out working in single cantilever flexural conditions.

#### 2.3.6. Color Measurements

With the aim of obtaining information about the color of the samples, a Konica CM-3600d Colorflex-DIFF2 colorimeter, from Hunter Associates Laboratory, Inc. (Reston, Virginia, USA), was used for the color measurement. Color indexes (L*, a*, and b*) were measured according to the following criteria: L* is the lightness and changes from 0 to 100; a* stands for the green (a* < 0) to red (a* > 0) color coordinate, while b*, represents the blue (b* < 0) to yellow (b* > 0) color coordinate. Measurements were done in quintuplicate.

#### 2.3.7. Water Uptake Characterization

The evolution of water absorption was studied using injection-molded samples of 4 × 10 × 80 mm^3^, which were immersed in distilled water at 23 ± 1 °C. The samples were taken out and weighed weekly using an analytical balance with a precision of ±0.1 mg, after removing the residual water with a dry cloth. The evolution of the water absorption was followed for a period of 15 weeks. Measurements were performed in triplicate.

#### 2.3.8. Statistical Analysis

To measure the significant differences among the samples were evaluated at 95% confidence level (*p* ≤ 0.05) by one-way analysis of variance (ANOVA) following Tukey’s test. Software employed for this propose was the open source R software (http://www.r-project.org).

## 3. Results

### 3.1. Mechanical Properties of BioHDPE-Hemp Fiber Composites

Mechanical characterization of BioHDPE composites with different proportions of hemp fiber provides relevant information about the properties and possible applications of the obtained composites. Table 2 shows the main mechanical parameters such as the elastic modulus (E), the maximum tensile strength (σ_max_) and elongation at break (ε_b_) of the BioHDPE/HF composites compatibilized with PE-g-MA.

BioHDPE shows typical tensile test values for this polymer, with an E modulus of 826 MPA and a tensile strength of 15.1 MPa. Like many other HDPE, elongation at break is extremely high, as the tensile test sample does not break during the test at 5 mm/min. These values are indicative of a material with great ductility, but with some stiffness. The values observed for BioHDPE are very similar to those reported by other authors and works [39].

The incorporation of different proportions of hemp fibers (HF) with PE-g-MA means an inflection point in the mechanical properties of green composites. Particularly, it can be observed how the addition of only 2.5 wt.% HF allows to obtain an E modulus of 1350 MPa and a tensile strength of 18.3 MPa, directly improving the stiffness values of neat BioHDPE. It should be remarked that the introduction of fillers and reinforcing agents normally reduces tensile strength values of the composites in comparison with the neat polymer matrix [40,41]. However, the addition of short hemp fibers in the BioHDPE avoids this reduction, generating even an improvement in tensile strength. In this context, authors such as Yomeni et al. [42] showed a similar behavior for low density polyethylene (LDPE) composites with a 30 wt.% of treated hemp fiber, obtaining a direct increase in the modulus and the resistance of the materials. On the other hand, the incorporation of this kind of fillers generates a clear negative effect, which is closely related to the lack of cohesion between the polymer and the filler, provoking a reduction of the ductile properties and, as a result, a direct reduction in elongation at break. With regard to 2.5 wt.% HF sample, an elongation at break of 9.1% is obtained, which is quite lower than that of BioHDPE (No break). These results are very similar to those obtained by several authors with the incorporation of different natural fibers in polymer matrices [43,44]. 

This behavior repeats in a linear way as the amount of hemp fiber in the polymer matrix increases. The incorporation of 5 wt.% and 10 wt.% of HF gave elastic modulus of 1410 and 1700 MPa and tensile strengths of 19.7 and 19.9 MPa, respectively. In the case of 5 wt.% of HF, the increase in E modulus is not significant, as it only increases 60 MPa in relation to the one obtained for the 2.5 wt.% composite. Nonetheless, an increase in tensile strength is observed from 18.3 to 19.7 MPa, which is an increase of more than 7%. In the case of the incorporation of 20% HF, a sample without PE-g-MA has been created in order to analyze the differences in mechanical properties. The sample without PE-g-MA shows an increase in modulus of 593 MPa in relation to the compatibilized sample. On the other hand, there is a reduction in tensile strength of almost 2 MPa. Finally, a clear reduction in elongation at break can be observed, going from 4.9% for the compatibilized sample to 4.0% for the non-compatibilized sample. The increase in the tensile modulus is typical of polymer-filled materials as the tensile modulus stands for the applied stress and the elongation in the linear/elastic region. Due to the dramatic decrease in elongation at break, the increase in tensile modulus is evident. Nevertheless, the good tensile strength of the Bio-HDPE composite with hemp fiber, which reveals a clear reinforcing effect, is worthy to note. Authors such as AlQahtani et al. [45] reported on how the incorporation of PE-g-MA in HDPE composites with natural fibers like date palm fiber, generates a clear improvement in terms of mechanical properties, obtaining quite balanced results, and les fragile materials. As it was studied in the previous work, the addition of PE-g-MA as a compatibilizer was a key element in terms of improving the affinity and ductility of BioHDPE composites with natural fibers [46]. As expected, the matrix continuity is reduced by the presence of the embedded hemp fibers which, in turn, decreases the overall cohesion and, therefore, the elongation at break is reduced. However, as discussed in previous work, due to the short fiber size, fibers can greatly reinforce the stiffness of the composites without generating large internal defects.

With regard to the incorporation of PE-g-MA into the composites, it can be observed how its introduction implies a clear improvement in the affinity between the polymer matrix and the natural fiber [47]. Finally, the sample that possesses 40 wt.% of HF provides very promising results with elastic modulus and tensile strength values of 5275 and 22.1 MPa, respectively. However, as it was expected, elongation at break is widely affected, giving a value of 2.2%. The addition of 40 wt.% of fiber implies a clear advantage from an environmental and economic point of view. Regarding the general values obtained for the 20 and 40 wt.% HF composites, other authors have reported on very similar results with different natural fibers. In particular, Mazur et al. [12] showed very similar results for HDPE/flax fiber composites. These results are strongly connected with the structure of the utilized fiber according to cellulose, hemicellulose and lignin contents. The cellulose contained in the fibers improves mechanical properties, although due to its hydrophilic nature, it does not successfully blend with the hydrophobic matrix. By contrast, lignin is an amorphous polymer, and it is less hydrophilic. Thus, it acts as a binder between cellulose and BioHDPE [48]. These mechanical properties are typical of a strong and tough engineered material.

In general, as it can be seen in Table 2, the addition of hemp fibers into BioHDPE provides a direct increase in the hardness of the composites. Initially, it can be observed how the introduction of 2.5 wt.% HF generates a great increase in hardness from 54.6 Shore D hardness for BioHDPE to 58.8 due to the hardening effect of the fibers. Regarding the composites with PE-g-MA and 5, 10, 20 and 40 wt.% of HF, a direct increment in the hardness values with the fiber content is observed, reporting values of 60.5, 62.1, 62.9 and 64.0, respectively. It can be seen how the blend without PE-g-MA (BioHDPE/20HF) shows a hardness value superior to that of the compatibilized composite, with a hardness value of 62.3. As it has been aforementioned, the introduction of PE-g-MA in the composites provokes a plasticizing effect. This is the reason why the non-compatibilized 20 wt.% HF sample has a superior hardness value compared to the compatibilized one [49]. Finally, the increase in hardness is directly related to the intrinsic hardness of the lignocellulosic fibers, which directly increases with the proportion of the fibers in the blend. 

Impact strength results show very interesting results in terms of some technical applications. Neat BioHDPE is a very ductile material with a relatively high impact strength (2.0 kJ/m^2^) obtained on notched test samples. This parameter is highly related to tensile strength and strain before fracture. HF samples of 2.5, 5 and 10 wt.% slightly reduce impact strength of the polymeric matrix, obtaining values of 1.7 and 1.8 kJ/m^2^, respectively. This reduction of impact strength values is linked with the appearance of internal stresses due to the low content in fiber, which is not enough to positively reinforce the composite [50]. Nonetheless, it is from 20 wt.% content of HF that a clear improvement in terms of impact strength is observed. 3.3 and 3.6 kJ/m^2^ for a 20 and 40 wt.% of hemp fiber content, respectively. In order to verify the aforementioned statements, the non-compatibilized 20 wt.% sample reduces its impact strength value down to 3.1, corroborating the improvement in fiber/matrix cohesion. This increase in impact strength is closely related to a higher amount and orientation of fibers.

The introduction of HF proportions superior to 20 wt.% in the BioHDPE matrix provides a clear increment in impact strength, with values between 3 and 4 kJ/m^2^. Particularly, the incorporation of 40 wt.% HF to the BioHDPE matrix improves impact strength by 80%. This increase is ascribed to the ability of those fibers to transfer loads longitudinally. This behavior is closely related to the fracture resistance theory. As was found in previous work [49], when these BioHDPE composites are brought under impact conditions, numerous microfractures appear in the first impact stages. Therefore, fibers enlarge along those microfractures, thus stopping their growth. Finally, and thanks to the incorporation of natural fibers, impact properties are considerably improved, as it can be observed in different WPC studies [51,52].

### 3.2. XRD Analysis

**Figure 2:**
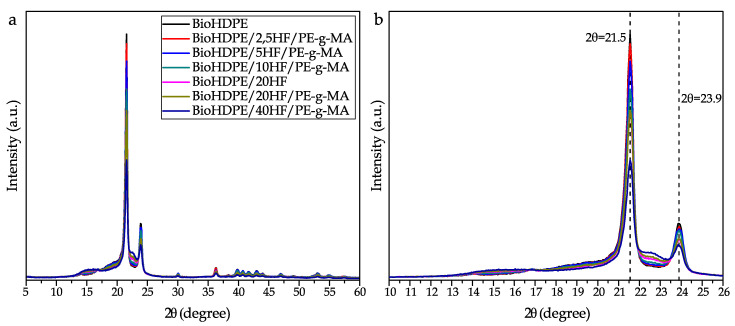
X-ray diffraction patterns for the BioHDPE/Hemp composites: (**a**) all range considered in the test; (**b**) detailed view of the main peaks.

Figure 2 shows the patterns obtained through XRD. BioHDPE is characterized for presenting an orthorhombic unit cell with two main peaks in 2θ = 21.56 and 2θ = 23.89, ascribed to the crystallographic planes (110) and (200). Moreover, lower intensity peaks appear in 2θ = 30.0 and 2θ = 36.2, which are relative to the (210) and (011) crystallographic planes. Additionally, there appear other low intensity peaks over 2θ = 40 as a consequence of the semicrystalline structure of HDPE [53]. The individual patterns of hemp fiber can be identified through literature analysis. In this case, wide and little pronounced peaks are remarked in 2θ = 15–16 and 2θ = 22.0, ascribed to the lattice planes (100) and (200). This spectrum present in hemp fibers is very similar to that obtained in other cellulose-based fibers [54]. The characteristic peaks of hemp can be seen in detail in Figure 2b, where it can be observed how the pattern of BioHDPE is slightly modified by the presence of the fibers. As it can be observed, those composites with higher content in fibers show higher intensity in the areas affected by cellulosic compounds. The results obtained here follow the same trend reported by Roumeli et al., who added hemp fibers into a HDPE matrix [34]. As a consequence of the incorporation of the fibers, the characteristic peaks of BioHDPE suffer a decrease in intensity. Liu et al. suggested that the introduction of fibers into the polymer provokes a distortion of the polymer structure, which results in the reduction of the peaks of the matrix in the test. [55]. Apart from the difference in intensity of the peaks, some authors have reported a modification of the position of the main planes of BioHDPE when different substances are added to the polymer matrix [56]. These position changes are normally related to a change in the distance between crystalline planes (d-spacing). However, in this case this phenomenon is not observed. These results show that crystalline regions are not widely present in hemp fibers, compared to amorphous regions [57]. This result is related to the fibers being moved and arranged along the fiber axis to impart better orientation, improving the mechanical properties. This may be due to the size of the hemp fibers, which do not greatly affect the crystallinity of the compound. Similar results where the position of the peaks did not change were reported by Farinassi et al., who incorporated spent coffee grounds in a HDPE matrix [58]. In this sense, the distance between crystalline lattices is 0.21 nm for the (110) planes, and 0.19 nm for (200) lattices, following Bragg’s equation [59]. Finally, it should be remarked that the addition of the compatibilizer (PE-g-MA), did not cause any significant effect. This can be seen in both (compatibilized and uncompatibilized) blends with 20 wt.% of hemp fiber, whose spectra is very similar. It could be ascribed mainly to the fact that PE-g-MA is PE-based, so it does not alter the internal composition of the composites, which are also PE-based.

### 3.3. Morphology of BioHDPE/Hemp Fiber Composites

**Figure 3:**
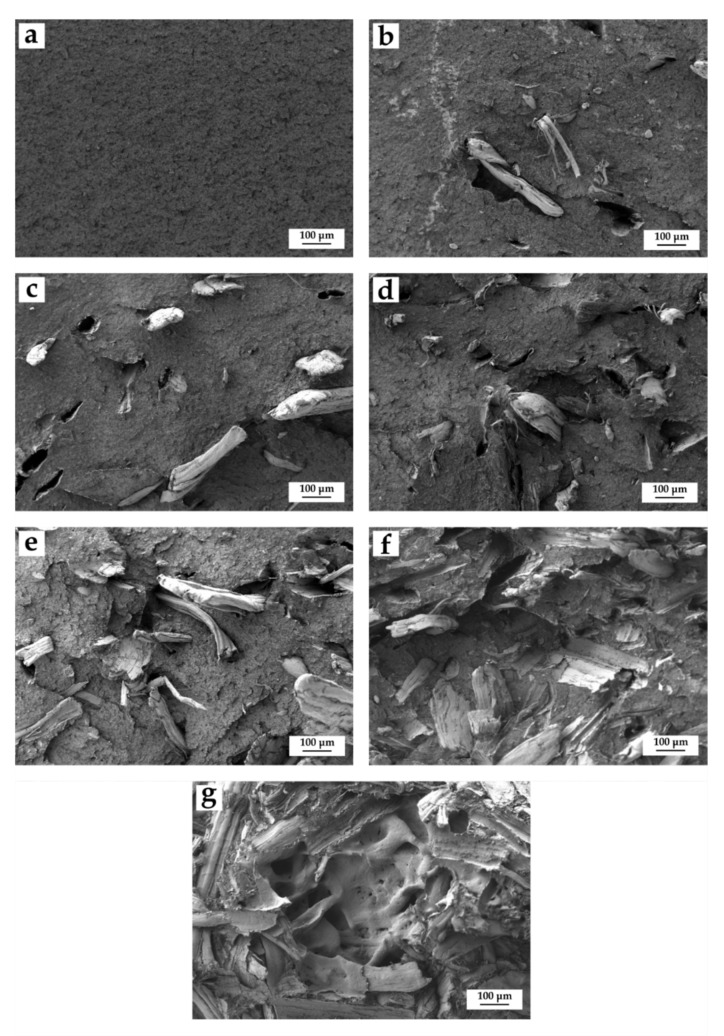
Field emission scanning electron microscopy (FESEM) images at 100x of the fracture surfaces of the BioHDPE/HF composites: (**a**) BioHDPE; (**b**) BioHDPE/2.5HF/PE-g-MA; (**c**) BioHDPE/5HF/PE-g-MA; (**d**) BioHDPE/10HF/PE-g-MA; (**e**) BioHDPE/20HF/PE-g-MA; (**f**) BioHDPE/20HF; (**g**) BioHDPE/40HF/PE-g-MA.

Figure 3 shows the FESEM images corresponding to the fracture surfaces of impact test injection-molded samples. Regarding neat BioHDPE, Figure 3a shows a ductile fracture, with a rough surface along all the observed the sample. The results shown in the image are typical for a polymer with great elongation at break and with high impact strength. These values correspond with the ones reported in previous works with the same BioHDPE [49].

In order to evaluate particle dispersion and the interaction in the fiber-matrix interface, the morphology of the fractured surfaces was observed. Figure 3b–g shows the morphology and distribution of the fibers in the composites with ascending content of hemp fiber in the BioHDPE matrix (from 2.5 to 40 wt.% of HF). In general, hemp fibers show very narrow gaps between the lignocellulosic filler and the matrix, which implies a good interaction. This excellent affinity between the fiber and the polymer provides a positive effect in the transfer of stresses and an improvement in toughness. Authors such as Mazzanti et al. [43] reported very similar results for hemp fibers in a PLA matrix, where treated and untreated fibers showed no gaps with the polymer matrix, providing a very promising fiber–polymer interaction. Moreover, in the case of high-content fiber samples, a greater concentration of fibers in the images can be observed (Figure 3e–g).

As the amount of hemp fiber increases within the matrix, higher concentration and saturation can be appreciated, ascribed to the volume of the filler in the green composite. Fibers are distributed quite homogeneously in the thermoplastic matrix, even in 20 and 40 wt.% HF samples, which implies a high volume content due to the low density of the lignocellulosic fibers [60]. Interestingly, despite the great fiber volume in the 40 wt.% HF sample, the interaction is good, corroborating the excellent mechanical results obtained for this blend. This suggests that there is an acceptable compatibility between the lignocellulosic particles and BioHDPE [7].

**Figure 4:**
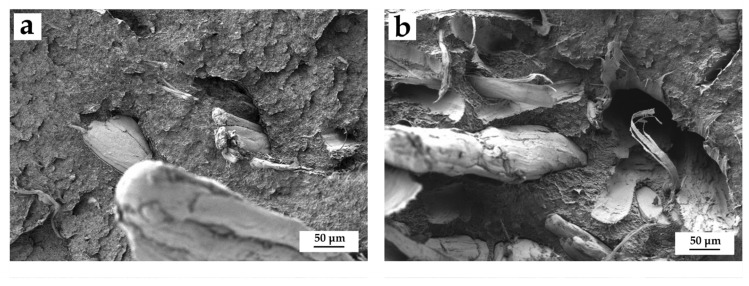
Comparative between Field emission scanning electron microscopy (FESEM) images at 250x of the comparison between composites with 20% HF with and without compatibilizers: (**a**) BioHDPE/20HF/PE-g-MA and (**b**) BioHDPE/20HF.

With regard to the incorporation of PE-g-MA, Figure 4 illustrates the real difference between compatibilized and uncompatibilized composites with 20 wt.% of HF. Figure 4a shows the composite with PE-g-MA, where a greater adhesion (smaller gap) between the fibers and the matrix can be appreciated. On the other hand, if the non-compatibilized sample is analyzed (Figure 4b), a greater presence of voids and gaps between fibers and the matrix is observed. This demonstrates the positive effect of the copolymer in the blend. Lima et al. [61] reported on a better interaction of BioHDPE/chitosan blends thanks to the compatibilization through PE-g-MA. Furthermore, this copolymer allows to improve particle dispersion and avoids the formation of aggregates with lignocellulosic fillers. All these effects support an increase in general mechanical properties.

The results obtained here verify that the affinity between HF and BioHDPE seems to be positive. Nonetheless, the addition of PE-g-MA further improves the fiber–polymer adhesion due to enhanced interactions and reduction in the width of the filler–matrix gaps.

### 3.4. Thermal Properties of BioHDPE/Hemp Fiber Composites

**Figure 5:**
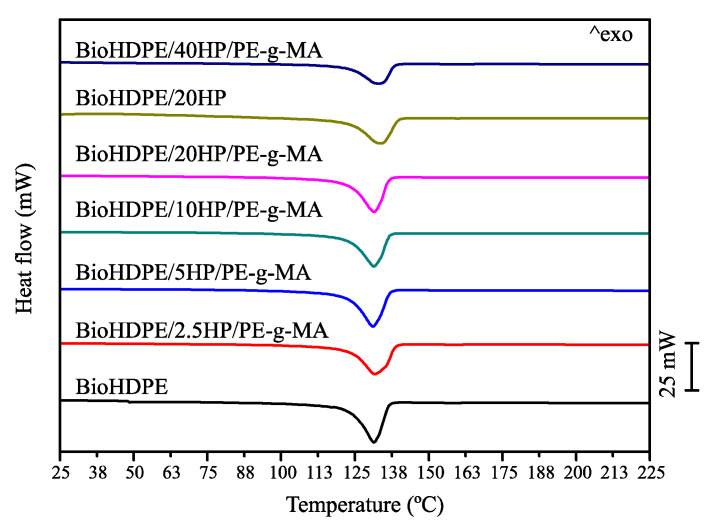
Differential scanning calorimetry (DSC) thermograms of BioHDPE/Hemp fiber composites.

Figure 5 shows the results obtained in differential scanning calorimetry (DSC) tests for the second heating cycle of BioHDPE/hemp composites. Additionally, the most relevant results are gathered in Table 3. The first parameter to analyze is the melting temperature of the different samples. It can be seen that the introduction of hemp in the blends does not produce differences in this parameter. The melting points of the blends are within the 131.3 °C and 133.9 °C range. These temperatures are similar to the ones obtained by Sewda et al., who proposed a melting point of 132 °C for HDPE with teak wood flour (TWF). This temperature was neither altered by the introduction of a compatibilizing agent such as HDPE-g-MAH [62]. Although the melting temperature is practically independent of the introduction of fibers, the melting enthalpy does vary due to the introduction of the fibers. This is ascribed to the diluting effect exerted by the addition of the fibers, which reduces the proportion of polymer chains that undergo the thermodynamic transition during melting [63].

With regard to crystallinity, a descending trend can be observed with the amount of HF introduced up until 10 wt.% HF, with a crystallinity degree of 58.0%. This tendency changes at greater fiber proportions, reaching a value of 68.8% for the composite with 40 wt.%, which is a similar value to the one obtained for neat BioHDPE. The introduction of loads can trigger two different phenomena related to the crystallinity degree: the first one is mobility restriction of the polymeric chains during the crystallization process, disrupting the ordering of the chains, thus preventing the crystallinity degree from increasing. An example of this phenomenon is shown by Silva et al., when he introduced fibers obtained from eucalyptus [64]. By contrast, in some cases the inclusion of fibers favors the formation of crystallization cores, leading to a higher degree of crystallinity, as it is reported in the work of Zhang et al., who introduced several fibers in a PLA matrix [65]. When this happens, the filler acts as a nucleating agent [66]. In this particular case, both effects occur simultaneously, depending on the amount of HF. At low concentrations of HF, the mobility restriction effect over polymer chains prevails. This effect changes at proportions higher than 10 wt.% HF, where the nucleating effect dominates and allows to increase the crystallinity degree, although never surpassing the crystallinity of neat BioHDPE. In relation to the effect of PE-g-MA, an increase in crystallinity is observed for compatibilized blends. In the non-compatibilized composites the nucleating effect is inhibited as a result of a poorer interaction between the filler and the matrix. Wang et al. reported similar results when comparing composites with the same amount of HF in compatibilized and uncompatibilized blends, observing an increase in crystallinity related to the presence of the compatibilizing agent [67].

DSC tests were carried out for every material and cooling speed, in order to determine the necessary time to reach certain relative crystallinity degree. From this information and the proposed equations, a linear regression was established to determine the slope and the intersection point of the obtained line, from which α and F(T) were calculated. These values are gathered in Table 4 [68]. The obtained results for the different blends showed a high correlation with the model, as it is demonstrated by the coefficient R^2^, which was superior to 0.92 in all the cases. For the same material there is an increasing trend of F(T) depending on the crystallinity degree of the sample. This parameter can also be related to the cooling speed necessary to reach a higher crystallinity degree in the sample. It is for this motive that a higher cooling speed is needed to achieve higher crystallinity degrees [69]. When the amount of hemp is increased in the polymeric matrix, F(T) also increases for every one of the relative crystallinity degrees considered. Yang et al. and Kuo et al. suggest that higher values of F(T) are related to an inferior crystallization speed due to the filler reducing the mobility of the polymeric chains [70]. As it was proposed beforehand, the introduction of the compatibilizer has a positive effect over the crystallinity degree of the sample. When compatibilized and non-compatibilized 20 wt.% HF samples are compared, the compatibilized sample shows a slower kinetic. Regarding the α value, it diminishes with the amount of hemp. Moreover, it slightly varies depending on the crystallinity degree. This low variability suggests that the crystallization mechanism does not vary during the non-isothermal cooling process [70].

**Figure 6:**
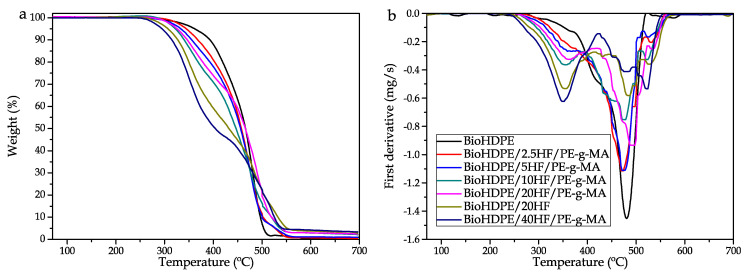
Thermal degradation of BioHDPE/Hemp fiber composites: (**a**) thermogravimetric (TGA) curves and (**b**) first derivative (DTG) curves.

Concerning the thermal stability of the BioHDPE/hemp composites, thermogravimetric diagrams are presented in Figure 6, while Table 5 gathers the main thermal parameter related to this test. Note how the incorporation of PE-g-MA notably improves thermal stability. If the compatibilized and non-compatibilized 20 wt.% HF composites are compared, the composite without PE-g-MA degrades at lower temperatures. This factor is highly related to the lack of affinity between the filler and the matrix, which stands for a poorer thermal stability. Two different behaviors can be observed in the degradation profiles of the samples. BioHDPE presents a curve with a single step, due to polyethylene being formed by big molecules, which blocks the volatilization process. This leads to the thermal scission of the polymeric chains at higher temperatures. According to Ueno et al., this process occurs with greater intensity at 450 °C. In this work, the maximum degradation temperature is located at 480 °C [71]. The introduction of the fibers provokes a change in the thermal degradation. These fibers have a great content in cellulose (44.5%), hemicellulose (32.8%) and lignin (22.0%) [72]. These compounds have a wide degradation range that is inferior to the degradation temperature of BioHDPE. Hemicellulose degrades at 220–315 °C, cellulose at 300–400 °C and la lignin in the temperature range 150–900 °C [73]. As a result, the degradation process of the composites is a combination of the compounds present in hemp fibers and BioHDPE. In Figure 6b a curve with a wide degradation range can be observed, which starts at 200 °C with the degradation of lignin. The presence of hemp makes an additional peak to appear in the 350–360 °C range, ascribed to the degradation of cellulose (T_deg1_). This behavior is common in WPC as it was reported by Jeske et al. [74]. Referring to the second degradation peak T_deg2_ at 480 °C, which corresponds to the degradation of the polymeric chains, it suffers a delay as a consequence of the great content in fiber. This is because the degradation of the lignocellulosic compounds reduces the amount of available oxygen in the sample. As a result, the oxidative degradation process of BioHDPE occurs at a higher temperature (521 °C for the BioHDPE/40HF/PE-g-MA sample) [75]. Additionally, note that the incorporation of PE-g-MA provokes a noticeable improvement in the thermal stability of the composites. If the composites with 20 wt.% HF are evaluated, it can be appreciated how the non-compatibilized composite degrades at a temperature 9 °C inferior compared to the compatibilized one. This effect is closely related to a lack of interaction between the fiber and the matrix, which generates a loss in thermal stability. This phenomenon was also observed in the work of Wang et al., in which the effect of different compatibilizing strategies of wood flour with polypropylene are tested in the thermal degradation field [76]. Finally, the residual weight of the samples is influenced by the amount of fibers in the samples. BioHDPE showed a residual weight of 0.3%. This value increases up to 3.3% for the blend with 40 wt.% HF. Stevulova et al. reported that at 600 °C, hemp has residual mass of 5.1% in oxygen atmosphere conditions [72].

### 3.5. Thermomechanical Properties of BioHDPE/Hemp Fiber Composites

**Figure 7:**
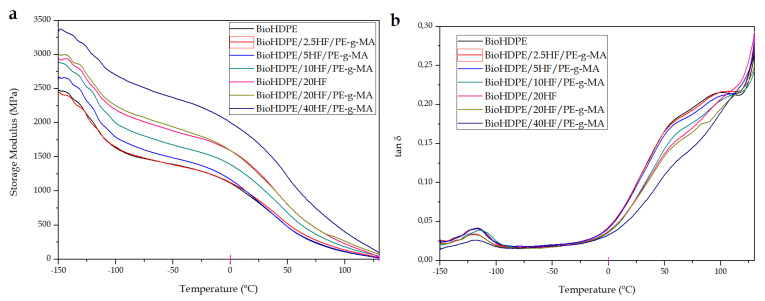
Thermomechanical properties of Bio-HDPE/Hemp fibers composites as a function of temperature: (**a**) storage modulus (E’) and (**b**) dynamic damping factor (tan δ).

Dynamic mechanical thermal characterization (DMTA) was used to evaluate the influence of temperature on mechanical behavior of BioHDPE/Hemp composites. In this sense, Figure 7 shows the thermomechanical behavior of the green composites. Particularly, Figure 7a represents the evolution of the storage modulus (E′) with temperature, while Figure 7b allows to evaluate the dynamic damping factor (tan δ) of the different composites with temperature. The maximum peak observed in the dynamic damping factor diagram is indicative of the glass transition temperature (T_g_) of the composites. On the one hand, it can be appreciated how BioHDPE exhibits a peak at −116 °C. This peak is directly ascribed to the glass transition of the material, which is related to the non-crystalline regions of polyethylene [77]. On the other hand, from 50 °C a second relaxation peak can be observed, which goes to 110 °C. This second peak is ascribed to an interlaminar shearing process [49]. These inflection points in the base material allow to thoroughly evaluate storage modulus values of the composites at different temperatures. In general terms, it can be seen how the incorporation of short hemp fibers imply a clear increase in the stiffness of the material all along the temperature range. Particularly, as the amount of fiber increases in the blends, the stiffness of the material also becomes higher. These results coincide with the aforementioned statements in mechanical and morphological properties.

To profoundly analyze the results obtained, Table 6 gathers the T_g_ values and storage modulus values at different temperatures for all the composites. The dynamic thermomechanical behavior of BioHDPE was defined by a E ′ value of 2460 MPa at −145 ° C. In the temperature range between −100 ° C and 0 °C, the storage modulus progressively diminished down to 1100 MPa. This decrease in mechanical stiffness relates to the glass transition of the material. Moreover, the storage modulus decreased even more due to the softening of the polymeric matrix [39]. Except for the 2.5 wt.% sample, the incorporation of the short hemp fibers provokes a clear increase in the rigidity of the composites in all the temperature range. In particular, it can be observed how for the 40 wt.% HF blends, values of 3350 MPa, 2100 MPa and 750 MPa at −145, 0 and 75ºC are obtained, respectively. These values are quite superior to the ones reported for BioHDPE, corroborating the increase in stiffness provided by those composites. Agüero et al. [17] reported very similar results in terms of rigidity for PLA composites with short flax fibers in which 20 wt.% of fiber proportion achieved storage modulus almost twice as high as the modulus of neat PLA. On the other hand, the incorporation of PE-g-MA as a compatibilizing agent reveals a behavior in accordance with the previously commented results. The incorporation of this copolymer to the blend causes a reduction in the stiffness of the composites in favor of an improvement in terms of ductile properties. Additionally, it should be noted that the presence of PE-g-MA does not alter the value of T_g_ between the compatibilized and non-compatibilized 20 wt.% HF blends.

With regard to T_g_ values of the composites with higher content in hemp fiber, they exhibit a slight reduction of approximately 2–4 °C. In particular, the incorporation of 20 wt.% HF reports the highest decrease. This effect is related to a modification of chain mobility due to an improvement in polymer–fiber interactions [78]. Finally, the incorporation of up to 40 wt.% short hemp fibers generates green composites with a great stiffness all over the temperature range. These results verify what has been observed up until this moment in mechanical properties. Thus, giving great application to those materials in fields where high stiffness is demanded.

### 3.6. Color Measurement and Visual Appearance of the Green Composites

**Figure 8:**
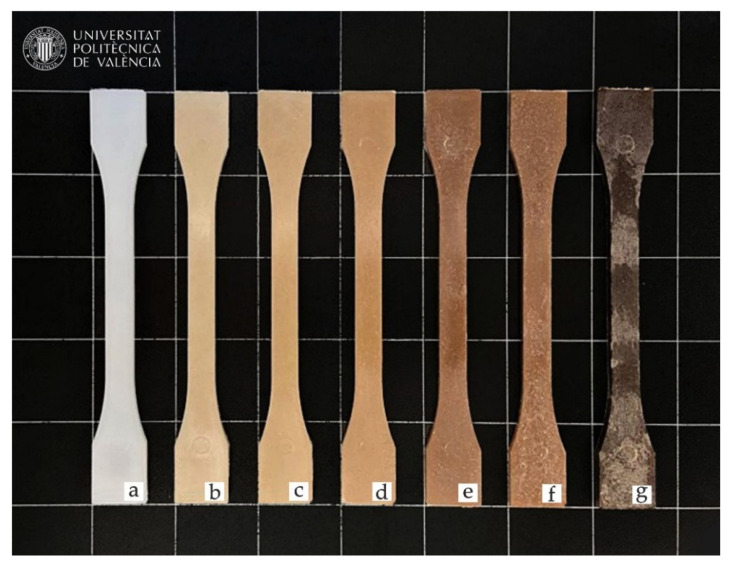
Visual appearance of the samples: (**a**) BioHDPE; (**b**) BioHDPE/2.5HF/PE-g-MA; (**c**) BioHDPE/5HF/PE-g-MA; (**d**) BioHDPE/10HF/PE-g-MA; (**e**) BioHDPE/20HF/PE-g-MA; (**f**) BioHDPE/20HF; (**g**) BioHDPE/40HF/PE-g-MA.

Colorimetric results obtained in the different composites after the fabrication process are shown in Figure 8, while color coordinates of the CIELab chromatic space are found in Table 7. In general, the introduction of natural fibers allows to obtain polymers with a wood-like appearance, which are normally called wood plastic composites (WPC). This is due to hemp fibers providing a brownish color to the composites. The change in color from the characteristic White of BioHDPE (L* = 68.7, a*= −2.0 y b* = −6.0), to brown occurs more intensely as the content in hemp fiber increases. Just with the incorporation of 2.5 wt.% of hemp fiber, color coordinates a* and b* already suffered an increase, which implies that red and yellow tonalities increase, respectively, resulting in brownish colors. a* and b* values are increased up to L* = 52.1, a* = 5.5 and b* = 20.1 for the 10 wt.% HF sample, providing a darker brown shade to the composite. Thus, colors similar to natural Woods are obtained, such as oak or eucalyptus woods [79,80]. The progressive color change effect in the samples is not only linked with the proportion of fibers. As it has been aforementioned, hemp fibers are compounded by different cellulose-based compounds with an onset degradation temperature of 150 °C for lignin. For the extrusion and injection processes, temperatures of 155 °C and 160 °C are used, respectively. As a result, during processing, lignin suffers a slight degradation that turns the sample into a darker color, especially to the 40 wt.% HF composite. Römer et al. analyzed the effect of applying 160 °C and 240 °C temperatures to a lignocellulosic compound, particularly, eucalyptus wood. These temperatures provoked L*, a* and b* parameters to decrease in all cases [81]. This darkening suggests that hemp has undergone a slight degradation during processing. Nonetheless, the mechanical properties of the analyzed samples showed a tensile strength of 22.1 MPa for the BioHDPE/40HP/PE-g-MA composite.

### 3.7. Water Uptake Characterization

**Figure 9:**
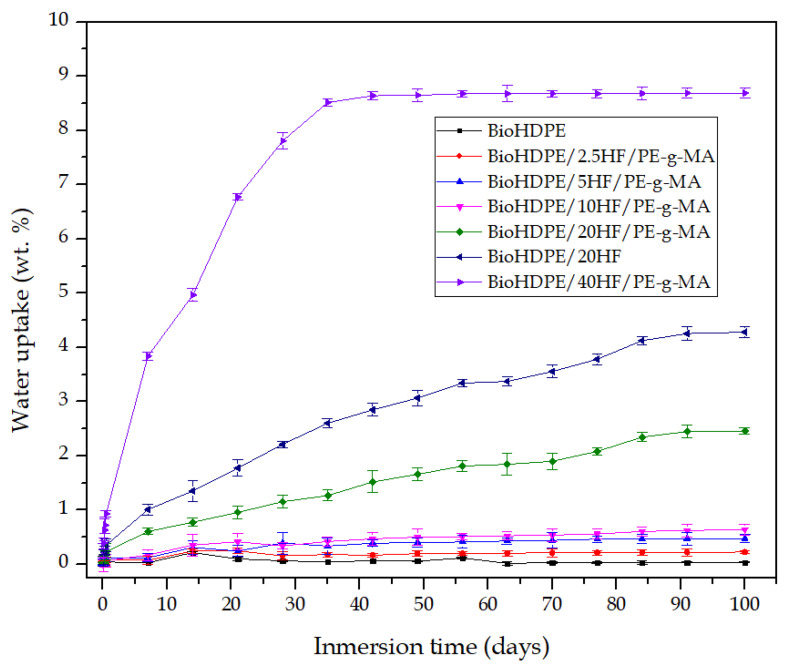
Water uptake of BioHDPE/Hemp fiber composites.

In general, wood plastic composites have the main drawback of containing a high proportion of lignin, cellulose and hemicellulose. These compounds are highly hydrophilic, which is not positive for certain industries and applications, as they are very sensitive to moister and water uptake. As a result, one of the main disadvantages of green composites is their tendency to absorb water. Figure 9 shows the evolution of water absorption of injection-molded pieces during 15 weeks of water immersion.

Neat BioHDPE barely absorbed any water, showing an asymptotic value at approximately 0.05 wt.%. This behavior is due to the highly non-polar nature of BioHDPE, which makes it a hydrophobic polymer with poor affinity for water (a polar solvent). Jorda-Reolid et al. [41] observed a similar water absorption diagram for BioHDPE. When incorporating hemp fiber and PE-g-MA into the structure of BioHDPE, the water absorption capacity of the blends increases with the content in hemp fiber. Hemp fiber samples of 2.5, 5 and 10 wt.% present maximum absorption values at 100 days between 0.25 and 0.5 wt.%. 20 wt.% HF blend with PE-g-MA increases water absorption up to 2 wt.% approximately, while 40 wt.% HF sample drastically augments it to 8.5 wt.%. This increase in water absorption is ascribed to the lignocellulosic nature of hemp fiber, which means it has highly polar compounds such as hemicellulose, cellulose, lignin and pectin, with oxygen-based functionalizations (hydroxyl groups) [82]. These compounds have great polarity, thus ensuring affinity for water. As a result, water absorption over time increases in relation to neat BioHDPE. As expected, the higher the HF content, the higher the uptake of water. Fang et al. [83] also reported the ability of BioHDPE/HF composites to retain water on their structure, achieving values of 11 wt.% of water retention. BioHDPE/20HF sample without PE-g-MA exhibited higher water absorption than BioHDPE/HF20. This could be related to the great proportion of PE-g-MA that this blend possesses (16 phr). PE-g-MA presents certain affinity for water due to its maleic anhydride functionalization [84], although it is not as water-absorbent as HF, which has several highly hydrophylic oxygen based groups. This fact makes that the absorbed water in relation to the weight of the simple diminishes in comparison with BioHDPE/20HF.

From these results, it can be deduced that BioHDPE/HF composites have great capacity to absorb water. Nonetheless, they can maintain a low degree of water absorption when the HF concentration does not surpass 10 wt.%, which is an interesting property considering applications where a highly hydrophilic behavior is not convenient [85].

## 4. Discussion

This work demonstrates that short hemp fibers derived from industrial and agri-food wastes can be efficiently used as new reinforcing elements in totally biological BioHDPE parts prepared by conventional industrial processes such as injection molding. Regarding mechanical properties, the increase in the fiber content up to 40 wt.% HF, notably increased Young’s modulus, going from 826 MPa for neat BioHDPE to 5275 MPa for BioHDPE/40HF/PE-g-MA. As it was expected, the incorporation of hemp fibers provokes a clear reduction in elongation at break of the composites as their content increases within the matrix. Nonetheless, this progressive increase provides an improvement in tensile strength, obtaining a value of 22 MPa for the 40 wt.% HF composite. These results verify the reinforcing effect that those natural fibers provide to a polymer matrix. FESEM images allow to evaluate more profoundly the correct distribution of the fibers along the matrix, corroborating the mechanical results thanks to a good adhesion between filler and matrix. This effect is enhanced by the presence of PE-g-MA. With regard to thermal properties, it should be remarked the variation of crystallinity in the composites depending on the fiber content. The sample with 10 wt.% HF obtained the highest crystallinity, with values very close to those of neat BioHDPE. In this sense, observing the crystallization kinetic results, higher F(T) values are observed for higher % of fiber for each one of the relative crystallization degrees considered. With respect to colorimetry, the introduction of hemp fibers gives the composites a brownish color, providing them with a wood-like appearance. Finally, as expected, BioHDPE/HF composites possess a great water absorption capability. However, they can maintain a low water absorption degree when the proportion of fiber does not surpass 10 wt.%, which turns to be an interesting property considering the applications where a highly hydrophilic behavior is not convenient.

## 5. Conclusions

All in all, the obtained results demonstrate that it is possible to obtain functional wood plastic composites with a high renewable content, even with a 40 wt.% of hemp fiber. The analyzed composites showed excellent properties, favoring the production of new environmentally friendly materials and supporting the circular economy concept. Apart from providing excellent mechanical properties in terms of stiffness, good compatibility and thermal stability; these composites have a great cost effective advantage, reducing the cost of the materials thanks to the addition of fibers. This study demonstrates the viability and the potential of incorporating great proportions of hemp fiber to develop new green composites with added value. Moreover, the ability of PE-g-MA as a compatibilizing agent is also proved. Thus, a new investigation route is opened from which new polymer matrices, compatibilizers and treatments on fibers could be evaluated.

## Figures and Tables

**Table 1:** Summary of compositions according to the weight content (wt.%) of Bio-HDPE and different proportions of Hemp fiber (HF) and PE-g-MA.

Code	BioHDPE (wt.%)	HF (wt.%)	PE-g-MA (phr)
BioHDPE	100	0	0
BioHDPE/2.5HF/PE-g-MA	97.5	2.5	0.25
BioHDPE/5HF/PE-g-MA	95	5	0.5
BioHDPE/10HF/PE-g-MA	90	10	1
BioHDPE/20HF/PE-g-MA	80	20	2
BioHDPE/20HF	80	20	0
BioHDPE/40HF/PE-g-MA	60	40	4

**Table 2:** Summary of mechanical properties of the BioHDPE-hemp fiber composites in terms of tensile modulus (E), maximum tensile strength (σ_max_), elongation at break (ε_b_), Shore D hardness and impact strength.

Code	E (MPa)	σ_max_ (MPa)	ε_b_ (%)	Shore D Hardness	Impact Strength (kJ/m^2^)
BioHDPE	826 ± 7 ^a^	15.1 ± 0.9 ^a^	NB	54.6 ± 1.7 ^a^	2.0 ± 0.2 ^a^
BioHDPE/2.5HF/PE-g-MA	1350 ± 50 ^b^	18.3 ± 0.5 ^b^	9.1± 0.5 ^a^	58.8 ± 1.9 ^a^	1.7 ± 0.1 ^a^
BioHDPE/5HF/PE-g-MA	1410 ± 22 ^b^	19.7 ± 0.2 ^b^	7.4 ± 0.3 ^a^	60.5 ± 2.0 ^b^	1.8 ± 0.0 ^a^
BioHDPE/10HF/PE-g-MA	1700 ± 12 ^c^	19.9 ± 0.7 ^b^	6.8 ± 0.4 ^b^	62.1 ± 1.2 ^b^	1.8 ± 0.1 ^a^
BioHDPE/20HF/PE-g-MA	2624 ± 75 ^d^	20.6 ± 0.6 ^b^	4.9 ± 0.5 ^b^	62.9 ± 0.5 ^b^	3.3 ± 0.1 ^b^
BioHDPE/20HF	3217 ± 250 ^e^	18.9 ± 0.9 ^b^	4.0 ± 0.4 ^c^	63.2 ± 0.9 ^c^	3.1 ± 0.3 ^b^
BioHDPE/40HF/PE-g-MA	5275 ± 150 ^f^	22.1 ± 0.3 ^c^	2.2 ± 0.2 ^d^	64.0 ± 1.0 ^c^	3.6 ± 0.2 ^b^

NB: Not Break, a–f Different letters in the same column indicate a significant difference among the samples (*p* < 0.05).

**Table 3:** Main thermal parameters of the composites with different amounts of hemp fiber in terms of melting temperature (T_m_), normalized melting enthalpy (ΔH_m_) and degree of crystallinity (*χ*_*c*_).

Samples	T_m_ (°C)	ΔH_m_ (J/g)	*χ*_*c*_ (%)
BioHDPE	131.3 ± 0.5 ^a^	202.0 ± 1.4 ^a^	68.9 ± 1.1 ^a^
BioHDPE/2.5HF/PE-g-MA	131.8 ± 0.4 ^a^	179.0 ± 1.2 ^b^	62.7 ± 1.1 ^b^
BioHDPE/5HF/PE-g-MA	131.8 ± 0.5 ^a^	177.4 ± 1.3 ^b^	63.7 ± 1.3 ^b^
BioHDPE/10HF/PE-g-MA	131.6 ± 0.3 ^a^	153.0 ± 1.4 ^c^	58.0 ± 1.4 ^b^
BioHDPE/20HF/PE-g-MA	131.3 ± 0.4 ^a^	144.8 ± 1.2 ^c^	61.8 ± 1.2 ^b^
BioHDPE/20HF	133.9 ± 0.5 ^a^	139.6 ± 1.3 ^d^	59.6 ± 1.4 ^b^
BioHDPE/40HF/PE-g-MA	132.9 ± 0.2 ^a^	120.9 ± 1.4 ^e^	68.8 ± 1.4 ^c^

a–e Different letters in the same column indicate a significant difference among the samples (*p* < 0.05).

**Table 4:** Non-isothermal crystallization kinetic parameters of BioHDPE/Hemp composites obtained by the Avrami–Ozawa model.

	Xt (%)	20	40	60	80
BioHDPE	F(T)	1.80	3.09	5.65	11.24
	α	1.52	1.63	1.63	1.45
	R^2^	0.93	0.92	0.93	0.96
BioHDPE/2.5HF/PE-g-MA	F(T)	1.88	3.15	5.60	11.52
	α	1.47	1.63	1.62	1.45
	R^2^	0.95	0.96	0.98	0.98
BioHDPE/5HF/PE-g-MA	F(T)	2.03	3.40	5.67	11.77
	α	1.42	1.54	1.64	1.52
	R^2^	0.95	0.93	0.95	0.98
BioHDPE/10HF/PE-g-MA	F(T)	2.91	3.55	5.92	11.89
	α	1.31	1.42	1.52	1.39
	R^2^	0.99	0.99	0.99	0.98
BioHDPE/20HF/PE-g-MA	F(T)	3.33	4.72	7.02	12.39
	α	1.27	1.39	1.49	1.29
	R^2^	0.95	0.95	0.96	0.99
BioHDPE/20HF	F(T)	5.39	7.41	10.09	15.06
	α	1.24	1.32	1.40	1.27
	R^2^	0.95	0.96	0.98	0.99
BioHDPE/40HF/PE-g-MA	F(T)	4.17	6.02	8.33	13.33
	α	1.15	1.30	1.33	1.21
	R^2^	0.94	0.94	0.95	0.97

**Table 5:** Main thermal degradation parameters of the composites with different amounts of hemp fiber in terms of: Temperature at mass loss of 5% (T_5%_), degradation temperature (T_deg_), and residual mass at 700 °C.

Samples	T_5%_ (°C)	T_deg1_ (°C)	T_deg2_ (°C)	Residual Weight (%)
BioHDPE	340.4 ± 1.4 ^a^	-	480.6 ± 2.2 ^a^	0.3 ± 0.1 ^a^
BioHDPE/2.5HF/PE-g-MA	344.2 ± 1.1 ^a^	362.4 ± 1.3 ^a^	470.9 ± 1.9 ^b^	0.4 ± 0.1 ^a^
BioHDPE/5HF/PE-g-MA	333.7 ± 1.2 ^b^	362.3 ± 1.1 ^a^	474.3 ± 1.6 ^b^	0.9 ± 0.3 ^a^
BioHDPE/10HF/PE-g-MA	323.6 ± 1.6 ^b^	352.1 ± 1.2 ^b^	473.1 ± 1.0 ^b^	2.2 ± 0.4 ^b^
BioHDPE/20HF/PE-g-MA	325.9 ± 1.5 ^b^	354.2 ± 1.0 ^b^	493.9 ± 0.9 ^c^	2.5 ± 0.2 ^b^
BioHDPE/20HF	307.2 ± 1.1^c^	352.7 ± 1.5 ^b^	484.3 ± 1.5 ^c^	2.9 ± 0.4 ^b^
BioHDPE/40HF/PE-g-MA	295.9 ± 1.3 ^c^	348.9 ± 1.1 ^b^	521.0 ± 2.1 ^d^	3.3 ± 0.3 ^c^

a–c Different letters in the same column indicate a significant difference among the samples (*p* < 0.05).

**Table 6:** Main thermomechanical properties of Bio-HDPE/Hemp fiber composites obtained by dynamic mechanical thermal analysis (DMTA).

Samples	E’ (MPa) at −145 °C	E’ (MPa) at 0 °C	E’ (MPa) at 75 °C	T_g_ (°C)
BioHDPE	2460 ± 45 ^a^	1110 ± 10 ^a^	230 ± 5 ^a^	−116.6 ± 1.2 ^a^
BioHDPE/2.5HF/PE-g-MA	2400 ± 32 ^a^	1120 ± 15 ^a^	280 ± 3 ^a^	−116.0 ± 0.9 ^a^
BioHDPE/5HF/PE-g-MA	2660 ± 50 ^b^	1170 ± 12 ^a^	245 ± 6 ^a^	−118.4 ± 1.0 ^b^
BioHDPE/10HF/PE-g-MA	2840 ± 45 ^b^	1390 ± 18 ^b^	360 ± 10 ^b^	−117.0 ± 0.9 ^b^
BioHDPE/20HF/PE-g-MA	2940 ± 49 ^c^	1600 ± 14 ^c^	450 ± 8 ^b^	−120.0 ± 0.9 ^b^
BioHDPE/20HF	3050 ± 55 ^c^	1650 ± 12 ^c^	455 ± 9 ^b^	−120.0 ± 0.7 ^b^
BioHDPE/40HF/PE-g-MA	3350 ± 68 ^d^	2010 ± 25 ^d^	750 ± 10 ^c^	−118.3 ± 1.0 ^b^

a–d Different letters in the same column indicate a significant difference among the samples (*p* < 0.05).

**Table 7:** Luminance and color coordinates (L*, a*, b*) of BioHDPE/Hemp Fibers.

Code	L*	a*	b*
BioHDPE	68.7 ± 0.4 ^a^	−2.0 ± 0.1 ^a^	−6.0 ± 0.2 ^a^
BioHDPE/2.5HF/PE-g-MA	61.4 ± 0.3 ^b^	0.2 ± 0.1 ^b^	13.1 ± 0.5 ^b^
BioHDPE/5HF/PE-g-MA	59.9 ± 0.3 ^b^	1.5 ± 0.1 ^b^	15.6 ± 0.6 ^b^
BioHDPE/10HF/PE-g-MA	52.1 ± 0.7 ^c^	5.5 ± 0.2 ^c^	20.1 ± 0.7 ^c^
BioHDPE/20HF/PE-g-MA	44.0 ± 0.1 ^d^	7.7 ± 0.1 ^d^	17.6 ± 0.1 ^c^
BioHDPE/20HF	46.3 ± 0.6 ^d^	7.8 ± 0.3 ^d^	16.0± 0.4 ^c^
BioHDPE/40HF/PE-g-MA	37.5 ± 0.5 ^e^	4.3 ± 0.1 ^e^	7.1 ± 0.3 ^d^

a–d Different letters in the same column indicate a significant difference among the samples (*p* < 0.05).

## Data Availability

Not applicable.
